# Evaluation of Methods for the Extraction of Spatial Muscle Synergies

**DOI:** 10.3389/fnins.2022.732156

**Published:** 2022-06-02

**Authors:** Kunkun Zhao, Haiying Wen, Zhisheng Zhang, Manfredo Atzori, Henning Müller, Zhongqu Xie, Alessandro Scano

**Affiliations:** ^1^School of Mechanical Engineering, Southeast University, Nanjing, China; ^2^Engineering Research Center of New Light Sources Technology and Equipment, Ministry of Education, Nanjing, China; ^3^Information Systems Institute, University of Applied Sciences Western Switzerland (HES-SO Valais), Sierre, Switzerland; ^4^Department of Neuroscience, University of Padova, Padua, Italy; ^5^Medical Faculty, University of Geneva, Geneva, Switzerland; ^6^UOS STIIMA Lecco – Human-Centered, Smart and Safe, Living Environment, Italian National Research Council (CNR), Lecco, Italy

**Keywords:** autoencoder (AE), muscle synergy, non-negative matrix factorization (NMF), independent component analysis (ICA), factor analysis (FA), principal component analysis (PCA)

## Abstract

Muscle synergies have been largely used in many application fields, including motor control studies, prosthesis control, movement classification, rehabilitation, and clinical studies. Due to the complexity of the motor control system, the full repertoire of the underlying synergies has been identified only for some classes of movements and scenarios. Several extraction methods have been used to extract muscle synergies. However, some of these methods may not effectively capture the nonlinear relationship between muscles and impose constraints on input signals or extracted synergies. Moreover, other approaches such as autoencoders (AEs), an unsupervised neural network, were recently introduced to study bioinspired control and movement classification. In this study, we evaluated the performance of five methods for the extraction of spatial muscle synergy, namely, principal component analysis (PCA), independent component analysis (ICA), factor analysis (FA), nonnegative matrix factorization (NMF), and AEs using simulated data and a publicly available database. To analyze the performance of the considered extraction methods with respect to several factors, we generated a comprehensive set of simulated data (ground truth), including spatial synergies and temporal coefficients. The signal-to-noise ratio (SNR) and the number of channels (NoC) varied when generating simulated data to evaluate their effects on ground truth reconstruction. This study also tested the efficacy of each synergy extraction method when coupled with standard classification methods, including K-nearest neighbors (KNN), linear discriminant analysis (LDA), support vector machines (SVM), and Random Forest (RF). The results showed that both SNR and NoC affected the outputs of the muscle synergy analysis. Although AEs showed better performance than FA in variance accounted for and PCA in synergy vector similarity and activation coefficient similarity, NMF and ICA outperformed the other three methods. Classification tasks showed that classification algorithms were sensitive to synergy extraction methods, while KNN and RF outperformed the other two methods for all extraction methods; in general, the classification accuracy of NMF and PCA was higher. Overall, the results suggest selecting suitable methods when performing muscle synergy-related analysis.

## Introduction

Muscle synergy theory assumes that the central nervous system (CNS) achieves a variety of motor tasks by combining a few sets of synergies rather than controlling each muscle individually. Although the hypothesis is debated, an increasing number of studies in human and animals using stimulation and behavior experiments have verified the theory in the last decades (d’Avella et al., 2003; Cheung, 2005; Torres-Oviedo et al., 2006). Muscle synergies are usually estimated from electromyogram (EMG) recordings according to corresponding models. Several synergy models have been proposed, such as the time-invariant model (Tresch et al., 2006), the time-varying model (D’Avella et al., 2006), and the space-by-time model (Delis et al., 2018; Hilt et al., 2018), while most studies extracted muscle synergies based on the time-invariant spatial model (Clark et al., 2010; Israely et al., 2018; Scano et al., 2019; Cheung et al., 2020) using the non-negative matrix factorization (NMF) (Lee and Seung, 2001), in which time-invariant synergies with fixed weights among muscles were modulated by time-varying activation coefficients. Commonly used factorization methods to extract spatial muscle synergies also include principal component analysis (PCA) (Ranganathan and Krishnan, 2012), factor analysis (FA) (Tresch et al., 2006; Kieliba et al., 2018), and independent component analysis (ICA) (Rasool et al., 2016). A few variants of ICA such as a combination of PCA and ICA (ICAPCA), fast ICA (fICA) and probabilistic ICA (pICA), and second-order blind identification (SOBI) were also applied in some studies (Tresch et al., 2006; Steele et al., 2015; Ebied et al., 2018).

Despite the availability of these factorization methods in the literature, the most appropriate method for muscle synergy extraction was not clearly defined (Rabbi et al., 2020). Several studies have compared the performance of factorization methods for the identification of spatial muscles or kinematic synergies under various scenarios. Tresch et al. (2006) evaluated the performance of five matrix factorization methods (i.e., FA, ICA, NMF, ICAPCA, and pICA) using simulated data. The results showed that the performance of factorization methods was affected by the signal characteristics and the noise type. They reported that ICAPCA and pICA were the best methods; FA, ICA, and NMF had similar performance, followed by PCA. By comparing the performance of three factorization methods (i.e., PCA, ICA, and NMF) in identifying kinematic and muscle synergies in human reaching data, Lambert-Shirzad and Van der Loos (2017) found that PCA and NMF had a comparable performance on both EMG and joint motion data and both outperformed ICA. When FA, ICA, and NMF were used to extract muscle synergies in locomotor tasks (Ivanenko et al., 2005), similar weighting coefficients and temporal structures were reported among the three methods. Ebied et al. (2018) evaluated three factors [i.e., muscle synergy sparsity, level of the noise, and the number of channels (NoC)] on the effects of factorization methods (i.e., PCA, ICA, NMF, and SOBI) in the extraction of spatial muscle synergy. They found that, although SOBI had a better performance when a limited NoC was available, NMF had the best performance when the NoC was higher. Furthermore, Rabbi et al. (2020) reported that NMF was the most appropriate extraction method in walking and running conditions by comparing four extraction methods (i.e., NMF, PCA, ICA, and FA). By comparing the results of previous studies, we learned that the performance of factorization methods was not always consistent under various settings and scenarios. Although these methods can reconstruct a high variance accounted for (VAF) with a proper number of synergies, they cannot represent non-linear relations between muscles in the extracted synergies, such as the agonist-antagonist relationships (Spüler et al., 2016). Besides, the studies were limited to synergy extraction analysis (input space); other desired tasks (task space), such as synergy-based classification, were less explored.

In the past decades, neural networks and deep learning-based methods have used surprisingly well in physiological and biomedical applications (Buongiorno et al., 2019b). Autoencoders (AEs) as a type of unsupervised neural network have also been used in myoelectric control and pattern recognition. Lv et al. (2018) reported that the AE was not sensitive to the electrode shift compared with time-domain and autoregressive features in classification tasks and achieved a lower classification error. Muhammad et al. also found that the stacked sparse AE outperformed the linear discriminant analysis (LDA) in hand motion classification whether in within-day or between-day analysis (Zia ur Rehman et al., 2018). In myoelectric control, Yu et al. (2019) achieved a promising wrist torque estimation under isometric contraction based on a stack-AE, with the potential of providing intuitive and dexterous control of artificial limbs (Vujaklija et al., 2018). In terms of synergy extraction, Spüler et al. (2016) first used AEs to extract muscle synergies and described the agonist-antagonist relationships among muscles using simulated data and real EMG data. They found that AEs had a significantly better fit to the data than other methods, including NMF, ICA, and PCA. De Feudis et al. (2021) used AEs to extract kinematic synergies and reported a comparable result with the PCA. By comparing with the NMF, the most frequently used method for muscle synergy extraction in the literature, Buongiorno et al. (2019a,2020) showed that the AEs outperformed the state-of-art synergy-based force/moment estimation methods at the expense of the EMG reconstruction quality. These studies described the potential of the AEs in myoelectric control as a bioinspired approach (Camardella et al., 2019) for muscle synergy extraction. To the best of our knowledge, few articles systematically compared and analyzed the performance of the AEs in muscle synergy extraction with other commonly used methods.

From the preliminary studies comparing several methods for extracting synergies (Tresch et al., 2006; Ebied et al., 2018), we noted a growing interest in the identification of the most suitable methods for extracting synergies and their properties also in the recent literature. These approaches include recent algorithms such as AEs (Spüler et al., 2016) and mixed-matrix factorization (Scano et al., 2022). In this study, we focused on comparing the capabilities of five factorization methods (i.e., PCA, ICA, FA, NMF, and AE) used for the extraction of spatial muscle synergy. Specifically, we evaluated the performance of AE in synergy extraction to the other four commonly used methods in the literature. We further explored the influence of the signal-to-noise ratio (SNR, i.e., noise) and the NoC on synergy extraction methods based on simulated data. Considering the wide usage of the synergy-based methods in myoelectric control, robotic control, and rehabilitation, we expanded toward comparisons between extraction methods to evaluate their performance in classification tasks. Muscle synergies extracted from a publicly available NinaPro dataset (Atzori et al., 2014, 2015), already employed previously for synergy extraction (Pale et al., 2020), were input into four classification algorithms, namely, K-nearest neighbor (KNN), LDA, support vector machine (SVM), and random forest (RF). Classification accuracy was used to assess the performance of each extraction method in the task space.

## Materials and Methods

**FIGURE 1:**
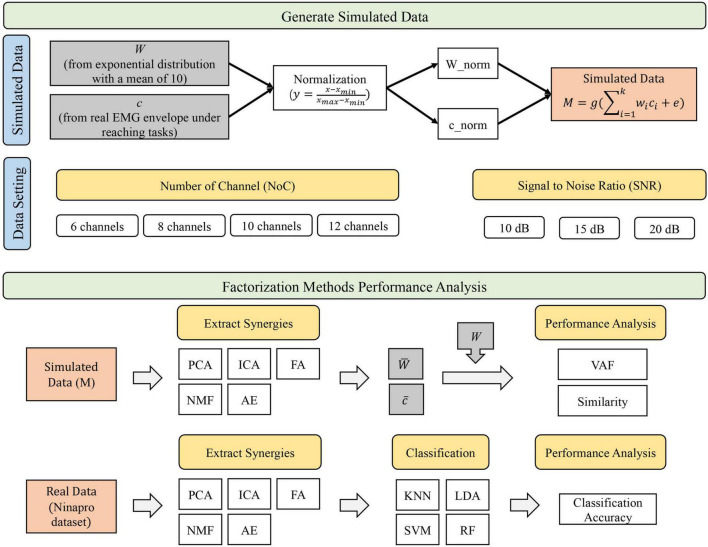
Overview of the study design.

An overview of the study design is reported in Figure 1. In this study, simulated data were generated to represent the ground truth of synergy vectors *W* and temporal coefficients *c* according to specific criteria (see “Simulated Data” section). Ground truth data also spanned across varying NoC and SNR. Then, the performance analysis was performed. We first evaluated the performance of synergy extraction methods with simulated data. In this step, synergies extracted using five methods were compared with the original ground truth (simulated synergies and temporal coefficients). The VAF and similarity were used as indices to assess the performance. Then, we assessed the performance of extraction methods in classification tasks using a publicly available dataset (NinaPro). Similarly, we first extracted the synergies from public data using five extracting methods. Extracted synergies were then input into four classification algorithms (i.e., KNN, LDA, SVM, and RF) to identify movements. Classification accuracy was used to assess the performance.

### Simulated Data

Simulated data were generated to evaluate the performance of the synergy extraction methods (Figure 1). According to previous studies, EMG activations are the combination of synergy vectors and activation coefficients, as shown in the following equation:


(1) $$\begin{array}{c} M=g\,\left(\sum_{i=1}^{k}w_{i}\,c_{i}+e\right) \end{array},$$

where *M* is an *m*-by-*n* matrix, indicating *m* muscles and *n* samples, *w*_*i*_ (*m*-by-1) is the *i*th synergy vector, and *c*_*i*_ (1-by-*n*) is the corresponding activation coefficient. *e* is the Gaussian noise matrix. *y* = *g*(*x*) is a threshold function with *y* = 0 for *x* < 0 and *y* = *x* for *x* ≥ 0, which ensures the non-negativity of the simulated data.

Our simulation began with the generation of the ground truth *W* and *c*. Synergy vectors *W* were randomly drawn from an exponential distribution [with a mean value of 10, similar to a previous study that reported that synergy vectors were roughly similar to the distribution observed in previous experimental data (Tresch et al., 2006)]. To hold the statistical properties of the EMG signals, the activation coefficients *c* were selected from the real EMG envelope signals randomly assigned from a set of reaching movements, in which ten upper limb muscle activities were recorded (Zhao et al., 2019). The raw EMG signals were preprocessed by moving the root mean square with a window size of 100 samples with 75% overlap, resampled to 1,000 sample points, and normalized to [0, 1] (*y* = (*x*−*x*_*min*_)/(*x*_*max*_−*x*_*min*_)). Each synergy vector was also normalized to have a unit norm. Then, we obtained simulated muscle activations according to Eq. 1, which were later used to evaluate the performance of extraction methods.

Meanwhile, to evaluate the performance of extraction methods in different settings, two types of constraints were set when generating simulated data. We first randomly generated a set of synergies with varying dimensions [NoC, i.e., number of muscles (*m*)] by fixing the number of synergies (*n*), i.e., a matrix with dimension *m*-by-*n*. This setting was used to evaluate the performance of extraction methods when a limited number of EMG signals were recorded. In this study, four types of channels (i.e., Ch6, Ch8, Ch10, and Ch12) were evaluated. This setting covered the studies in which 8–12 muscle recordings were measured in their experiments. When generating the ground truth, four synergies were fixed in this study because previous studies in upper limb reaching movements reported that four synergies were sufficient to explain most of the variability of muscle activations (Israely et al., 2017). We, in this study, remark that any reasonable number of synergies could be chosen in principle. Then, Gaussian noise was added to the original signal with a specified the signal-to-noise ratio [*SNR* = *10⋅lg*(*P*_*s*_/*P*_*n*_), *P*_*s*_ and *P*_*n*_ are signal and noise power, respectively] when each dataset was generated. Three types of noise (with SNR 10, 15, and 20 dB) were covered in this study. We finally generated 24,000 (20 × 4 × 3 × 100) trials that consisted of 20 datasets, and each dataset consisted of 100 trials for each setting.

### Extraction Methods

Although the computation process of these extraction methods is different, they can be represented by the linear combination of a set of synergy vectors and corresponding activation coefficients as follows:


(2) $$\begin{array}{c} M=c\cdot W+e \end{array},$$

where *M* represents muscle activations (the processed EMG) and *W* and *c* are muscle synergies and activation coefficients, respectively. Although sharing the same model, each extraction method imposes different constraints on the input signals and extracted synergies. PCA constrains *W* to be orthogonal, and the first component has the largest variance. Both PCA and FA assume that the data are from Gaussian distributions, while ICA is designed to analyze non-Gaussian data. The number of common factors that can be assessed using FA is limited by the degrees of freedom in the model, that is [(*m*−*k*)^2^−(*m* + *k*)] > 0 (e.g., when four common factors are identified, the data have at least eight dimensions). NMF can be used for both Gaussian and non-Gaussian data but imposes a non-negativity constraint on the components of the extracted synergies. PCA, FA, and NMF were performed using the Matlab functions *pca*, *factoran*, and *nnmf*, respectively. Singular value decomposition was used to identify the components in PCA. For FA, the weighted least-squares method was used to estimate the factor scores. Multiplication update rules proposed by Lee and Seung (2001) were applied in NMF. ICA was performed using the function *fastica* in the FastICA package (Hyvärinen and Oja, 1997; Hyvarinen, 1999).

**FIGURE 2:**
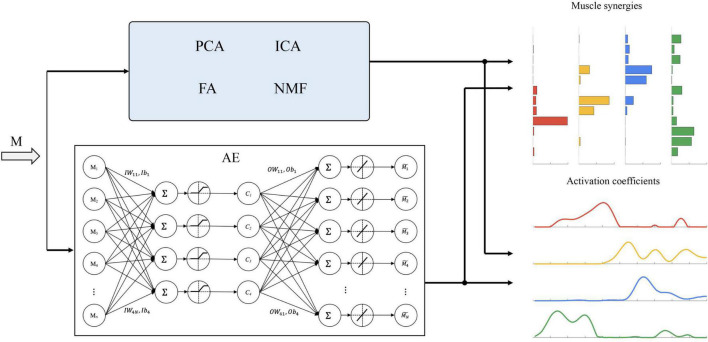
Schematic representation of the extraction methods considered in this study. Principal component analysis (PCA), independent component analysis (ICA), factor analysis (FA), and non-negative matrix factorization (NMF) follow a similar procedure for synergy extraction, which factorizes muscle activations (M) into a set of muscle synergies and corresponding activation coefficients. The topology structure of the autoencoders used in this study is also shown.

Autoencoders consists of two main parts, namely, an encoder that captures the representative features contained in the input data and a decoder that reconstructs the input. According to different internal structures, five types of AEs are proposed, namely, undercomplete AE, regularized AE, sparse AE, denoising AE, and variational AE (De Feudis et al., 2021). In this study, an undercomplete AE (denoted as AE below) was used to learn some non-linear coupling information among muscles. The topology structure of the undercomplete AE is shown in Figure 2.

The Matlab function *trainscg* was used to train the AE. The training process was based on the optimization of a cost function, *msesparse*, which measured the error between the input and the output. The transfer functions of encode and decode were *satlin* and *purelin*, respectively (Buongiorno et al., 2019a,^2020^).

We defined the weights and biases of the encoder (i.e., *IW* and *Ib*) and decoder (i.e., *OW* and *Ob*) (Figure 2). Muscle synergies are defined as *OW*, and activation coefficients *c* and reconstructed muscle activation $\tilde{M}$ are as follows:


(3) $$\begin{array}{c} \left\{ \begin{array}{c} c=I\,W\times M+I\,b\\ \tilde{M}=O\,W\times c+O\,b \end{array}\right. \end{array}$$

### Performance Analysis

Two indices were used to assess the performance of the employed extraction methods, i.e., (1) variance accounted for calculated by each extraction method under various settings and (2) the similarity between original synergy vectors (and activation coefficients) and the synergies identified using the extraction methods.

First, we decided to extract four synergies from the simulated data using each extraction method. This choice was followed based on a previous study: when comparing several algorithms for synergy extraction, Tresch et al. (2006) reported that, in all cases, the algorithms were examined using four basis vectors to reconstruct each data set. More recently, Ebied et al. (2018) also reported that, in all settings, the number of synergies was fixed to four. This choice was also in line with the ground truth dimensionality. Moreover, fixing the number of synergies allowed us to compare synergies that have low influence from merging or fractionation. The VAF from each extraction method was compared to assess the difference caused due to the varying settings.

Then, we matched the synergy vectors between the original simulated and the extracted ones by pairing together the two vectors from the original ground truth synergy vectors and the extracted, which has the largest absolute value of the dot product. In case that the largest value was negative, we reversed the sign of the synergy vectors identified using the extracting methods as in a previous study (Tresch et al., 2006). Then, the synergy vector similarity (SVS) and activation coefficient similarity (ACS) were computed by averaging the dot products among all matched synergy vectors and activation coefficients. We further calculated the principal angle (PA), which quantified the similarity between subspaces identified by original synergy vectors and the extracting methods. If the two subspaces are identical, the PAs between them will be zero.

Finally, to evaluate the influence of chance, 24,000 sets of synergy vectors and activation coefficients were randomly generated according to the same constraints used to generate the simulated data. We calculated and compared the similarity between the randomly generated synergy vectors and activation coefficients and the extracted ones.

### Classification Tasks

As a further step in our study that is linked to the promising results of muscle synergy-based applications in classification and intuitive prosthetic control (Jiang et al., 2009, 2012, 2014; Ma et al., 2015), we were interested in quantifying the classification accuracy that can be achieved with classification algorithms when synergies extracted with different methods were used as inputs. We questioned which synergy extraction method can be suggested for classification problems when using standard classification algorithms. The NinaPro dataset 1 (Atzori et al., 2014, 2015) was used as the dataset for this study. It includes 53 movements from 27 intact subjects with ten muscles from the upper limb. Eight electrodes were uniformly placed beneath the elbow at a fixed distance from the radio-humeral joint, labeled incrementally counterclockwise starting from the flexor carpi ulnaris muscle (Pale et al., 2020), while the other two were placed on the flexor and extensor muscles (Atzori et al., 2012). These muscles cover the main forearm muscle groups involved in upper limb reach-to-grasp movements. Considering the importance of wrist movements in daily living and implementation in prosthesis control (Ma et al., 2015), a set of wrist-related movements (Ninapro dataset 1, Exercise B, Movement 11–16) were considered as classification movements in this study (Atzori et al., 2014), i.e., wrist supination/pronation, wrist flexion/extension, and wrist radial/ulnar deviation.

We first separated the EMG signals for each movement and each trial from the dataset for each subject. In this study, each trial represents the movement in the positive or negative direction of one degree of freedom, such as wrist flexion or extension. According to previous studies (Jiang et al., 2014; Vujaklija et al., 2018), thus, one synergy was extracted from each trial, while a total of two synergies were extracted for each degree of freedom, basically representing flexion and extension. Then, all extracted synergies were used to train four commonly used classification algorithms, namely, KNN (with five nearest neighbors and Euclidean distance was used for distance measurement; Matlab function, *fitcknn*) (Ebied et al., 2018), LDA (Matlab function, *fitcdiscr*) (Zia ur Rehman et al., 2018), SVMs (with linear kernel function; Matlab function, *fitcecoc*) (Atzori et al., 2012), and RFs (with 50 weak learners; Matlab function, *TreeBagger*) (Atzori et al., 2014). The program performed a 5-fold cross-validation. The classification accuracy was computed to evaluate the performance of synergy extracting methods in the task space.

### Statistical Analysis

To test if extraction methods and different settings affected the VAF and the similarity of synergies and activation coefficients, a statistical analysis was conducted. One-way ANOVA was first used to test the effect of SNR and NoC on VAF identified by each extraction method. A *post hoc* test (*t*-test) was run to quantify the statistical difference among settings (three types of SNR and four types of NoC).

Furthermore, one-way ANOVA and the Tukey-Kramer *post hoc* test were used to examine statistically significant differences of similarity obtained with different settings. We then used one-way ANOVA and the Tukey-Kramer *post hoc* test to test if classification algorithms had a significant influence on the classification accuracy for each extraction method. Matlab R2020b was used for statistical analysis. The significance level was set at *p* = 0.05.

## Results

### Variance Accounted for Analysis

**FIGURE 3:**
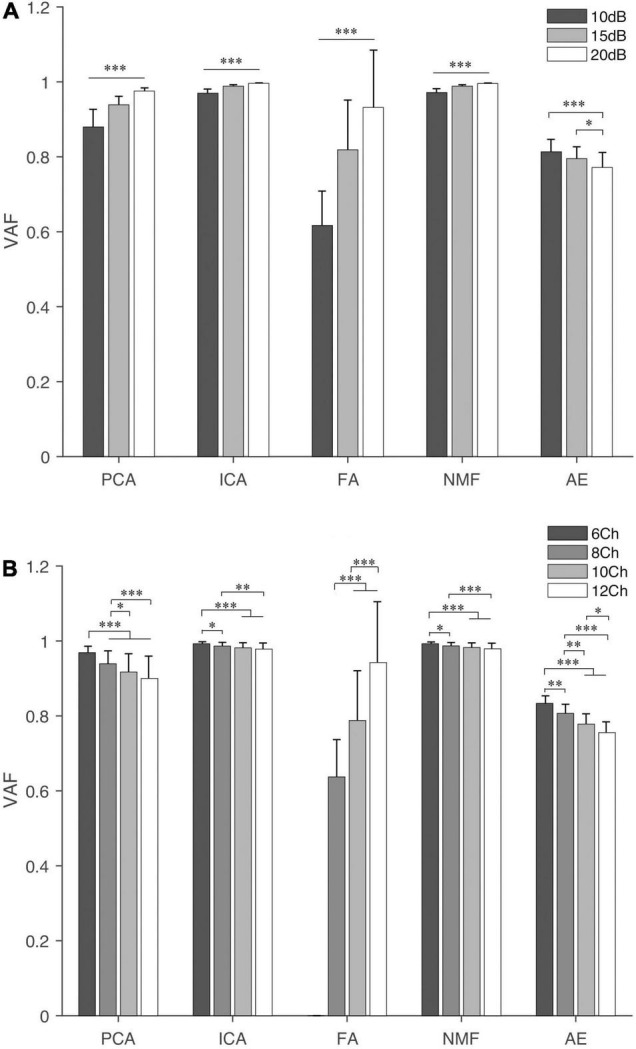
The variance accounted for (VAF) of the five extraction methods under different settings: SNR **(A)** and NoC **(B)**. For FA, when four synergies are extracted, at least eight muscles should be included. Thus, three bars are shown for FA. “^∗^”, “^∗∗^”, and “^∗∗∗^” indicate the significance levels are 0.05, 0.01, and 0.001, respectively.

Figure 3 shows the VAF of five extraction methods under different SNR and NoC. We observed that NMF and ICA had a higher VAF value under both settings (i.e., SNR and NoC), followed by PCA. AE and FA both had an average VAF lower than 0.8. Statistical analysis showed that both SNR and NoC had a significant influence on VAF (*p* < 0.001). The VAF of four commonly used methods (i.e., PCA, ICA, FA, and NMF) showed an increasing trend with the increase of the SNR (i.e., the decrease in the noise level) while the VAF of AE decreased with the increase of the SNR. In contrast, the VAF of PCA, ICA, NMF, and AE decreased with the increase of the NoC. VAF of FA showed an apparent increase with the increase of both factors.

In terms of SNR, the *post hoc* test showed that the difference was significant (*p* < 0.001) for any two different SNR settings except AE, in which the significance level between 10 and 15 dB was *p* = 0.128. In contrast, for different NoC settings, there was no significant difference when the NoC was 10Ch and 12Ch for PCA, ICA, and NMF. In terms of FA and AE, statistical differences were observed for any two different NoC settings.

### Synergy Similarity Analysis

**FIGURE 4:**
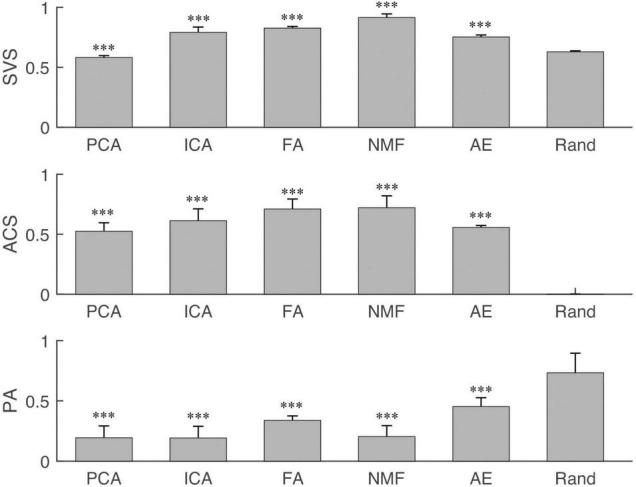
The synergy similarity of the extraction methods and random data. Three panels from top to bottom are synergy vector similarity (SVS), activation coefficient similarity (ACS), and principal angle (PA). The last bar in each panel “Rand” is the similarity between randomly generated data and the simulated data. The star shows the significance level between methods and Rand. “^∗∗∗^” indicate the significance levels are 0.05, 0.01, and 0.001, respectively.

**FIGURE 5:**
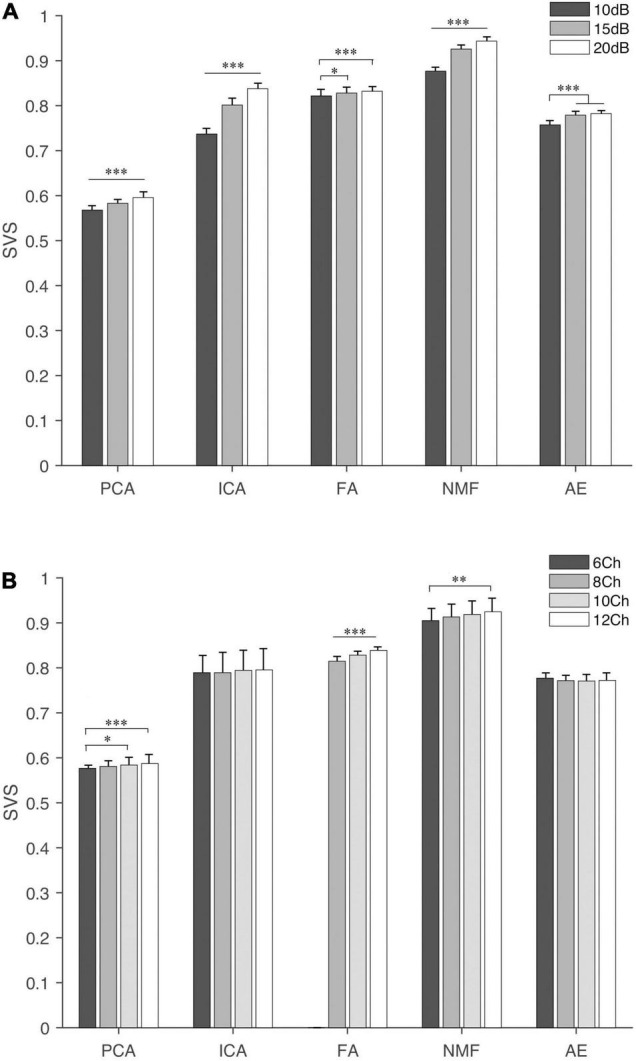
The SVS of extraction methods under different settings, **(A)** SNR and **(B)** NoC. The fine line on the bar is the standard error between trials. “^∗^”, “^∗∗^”, and “^∗∗∗^” indicate the significance levels are 0.05, 0.01, and 0.001, respectively.

**FIGURE 6:**
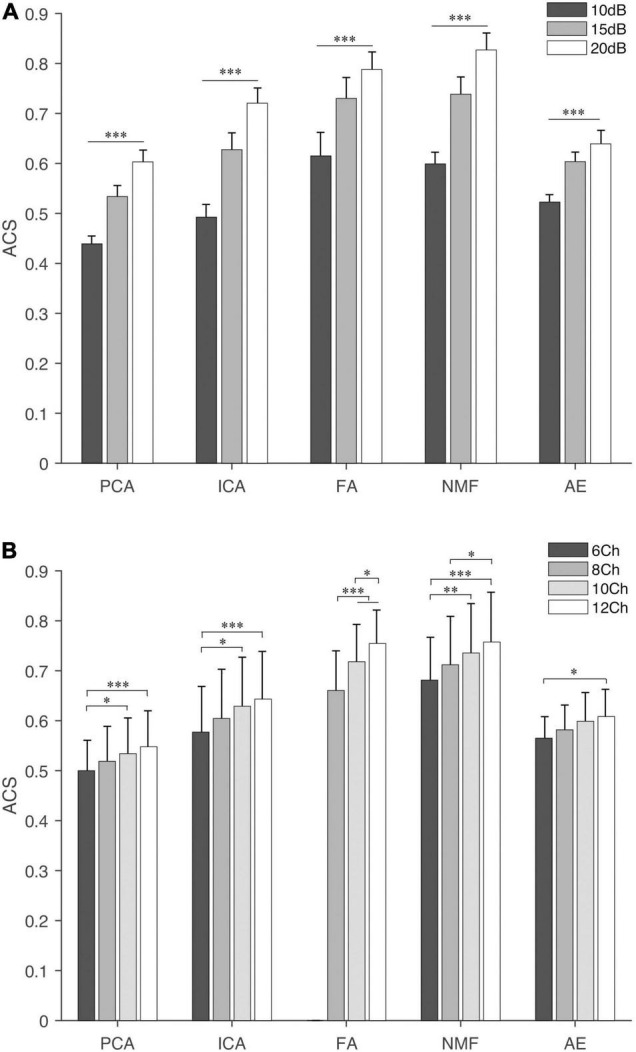
Activation coefficient similarity of extraction methods under different settings, **(A)** SNR and **(B)** NoC. The fine line on the bar is the standard error between trials. “^∗^”, “^∗∗^”, and “^∗∗∗^” indicate the significance levels are below 0.05, 0.01, and 0.001, respectively.

**FIGURE 7:**
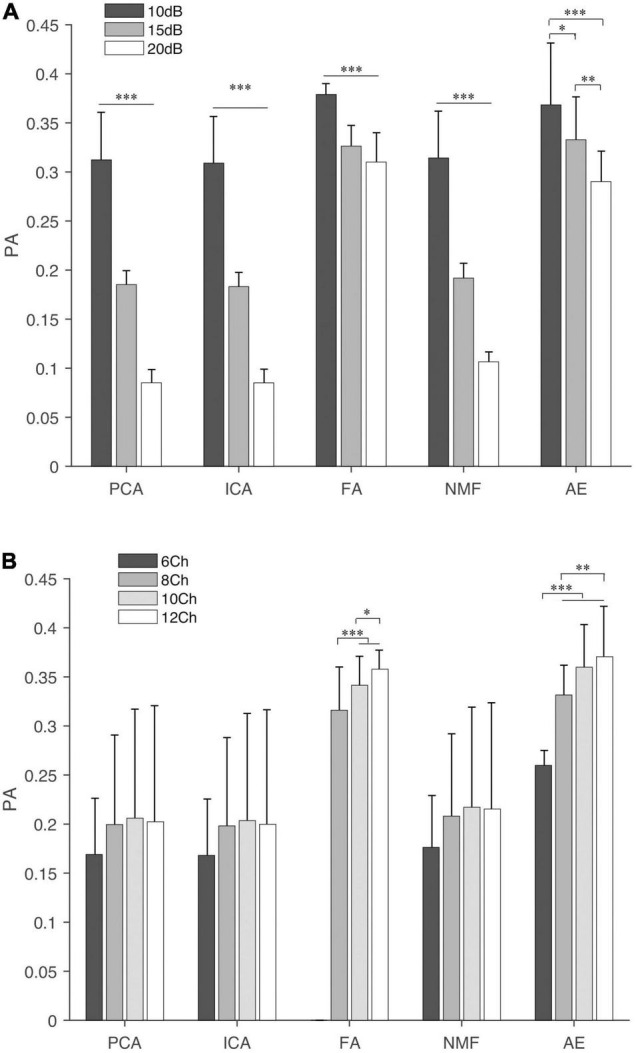
Principal angles of extracting methods under different settings, **(A)** SNR and **(B)** NoC. The fine line on the bar is the standard error between trials. “^∗^”, “^∗∗^”, and “^∗∗∗^” indicate the significance levels are 0.05, 0.01, and 0.001, respectively.

Similarity analysis among extraction methods is shown in Figures 4–7. First, SVS, ACS, and PA were significantly better (*p* < 0.001) than those obtained by random generation (Figure 4) except for the SVS calculated by PCA, which was significantly lower than the chance level (0.58 ± 0.016 vs. 0.63 ± 0.009).

The influence of different settings on the similarity and PA is shown in Figures 5–7. NMF and FA had higher SVS under both settings, followed by ICA and AE, while PCA had the lowest similarity value (Figure 5). Besides, SVS increased with the increase of SNR and NoC for each extraction method. The SNR had a significant influence (*p* < 0.001) on the SVS. The *post hoc* test showed that, when SNR was larger than 10 dB, there was no statistical difference in SVS for FA (*p* = 0.209) and AE (*p* = 0.283). In terms of the influence of NoC on SVS, a significant difference was observed among settings for FA. In contrast, there was no statistical difference among NoC settings for ICA (*p* = 0.801) and AE (*p* = 0.454). For PCA and NMF, when the NoC was higher than 6Ch, there was also no significant difference in SVS.

Similar results were observed in the ACS (Figure 6). NMF and FA had higher ACS under both settings, followed by ICA and AE, and PCA had the lowest similarity value. The SNR had a significant influence (*p* < 0.001) on the ACS. In contrast, the NoC had a different influence on ACS among extraction methods. ACS of FA was significantly influenced by the NoC (*p* < 0.001). For the other four extraction methods (i.e., PCA, ICA, NMF, and AE), in general, when the NoC was higher than 6Ch, there was no significant difference among different settings, while the ACS between 8Ch and 12Ch of NMF was *p* = 0.04.

For the PA (Figure 7), in general, FA and AE had a larger PA under both settings, followed by NMF, PCA, and ICA. SNR had a significant influence on five extraction methods (*p* < 0.001). NoC had no statistical influence on PA calculated by PCA (*p* = 0.142) and ICA (*p* = 0.159). The *post hoc* test showed that the PA of NMF was not affected by the NoC, while it affected the results of FA and AE.

### Classification Accuracy

**FIGURE 8:**
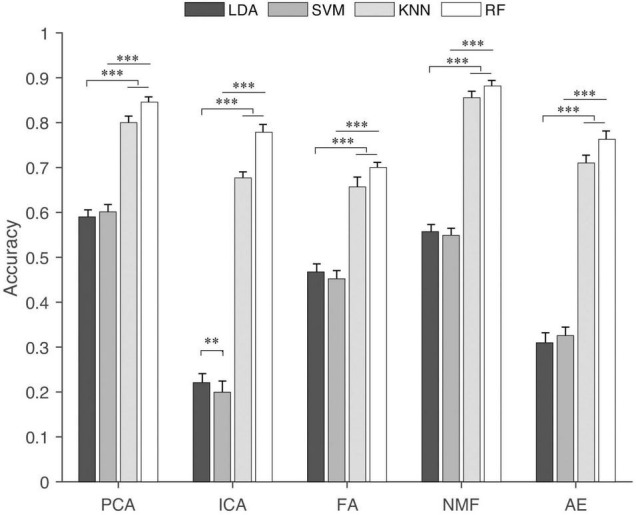
Classification accuracy of different classification algorithms for each synergy extraction method. “^∗∗^” and “^∗∗∗^” indicate the significance levels are 0.01 and 0.001, respectively.

The results of the classification accuracy of different classification algorithms based on the extracted synergies from five extracting methods are shown in Figure 8. In general, synergies extracted by NMF and PCA had higher classification accuracy. This was followed by FA and AE, and ICA had the lowest classification accuracy. For each extraction algorithm, RF and KNN had higher classification accuracy than LDA and SVM. Statistical analysis showed that classification algorithms had a significant influence (*p* < 0.001) on the classification accuracy for each extraction method. The *post hoc* test showed that the classification accuracy obtained by KNN and RF was significantly higher than that by LDA and SVM. When the synergies extracted by ICA were as input, LDA and SVM showed a statistically different classification accuracy.

## Discussion

In this study, we evaluated the performance of five synergy extraction methods using a set of simulated and experimental data. With respect to previous studies, this study introduced several novel aspects, including the performance analysis of an AE with the other four well-established synergy extraction methods under different settings and the coupling with classification tasks to link our results to real applications.

Several studies have used simulated and real data to evaluate the performance of commonly used factorization methods in spatial synergy extraction (Tresch et al., 2006; Ebied et al., 2018). However, these commonly used methods not only had specific constraints on input signals but also considered the variable reconstruction rate of the EMG signals as the only performance index. Besides, these methods did not incorporate the knowledge of the mechanical actions of muscles (Tresch et al., 2006), such as the agonist/antagonist activities and task performance (Spüler et al., 2016).

Among these methods, NMF is usually the most popular method for synergy extraction used in the majority of the studies. NMF imposes a non-negative constraint on the inputs (processed EMG signals) and outputs (synergies and activation coefficients). In a non-negative space, the basis vectors (extracted synergies) are not constrained to be orthogonal but are constrained to be independent (Lambert-Shirzad and Van der Loos, 2017). The non-negative property makes the extracted synergies particularly appropriate for clinical explanations because it reflects the non-negative nature of neural commands and muscle contraction.

Independent component analysis decomposes a multivariate signal into independent non-Gaussian signals by maximizing the statistical independence of the estimated components (Hyvärinen and Oja, 2000). ICA is designed to analyze non-Gaussian data and is substantially affected by the noise structure. Tresch et al. (2006) showed that ICA had a better performance when the signals were corrupted by constant variance Gaussian noise. However, the results of ICA depend heavily on the independency and data distribution of the latent variables (muscle synergies). Due to the difference of the simulated or experimental data in the current study from previous studies, this study showed a better performance using ICA. Besides, some studies (Tresch et al., 2006; Steele et al., 2015) compared the performance of several variants of the ICA such as pICA and PCAICA and reported that PCAICA was one of the most computationally efficient methods (Tresch et al., 2006). However, if the datasets with different muscle activations correlations were considered, similarity analysis dramatically changed (Steele et al., 2015). Thus, the results were limited to this study, and extensions to other implementations are required in future studies.

Both NMF and ICA extract independent components from the input data and have specific constraints on the inputs. Other methods employed in this study such as PCA and FA have instead different constraints on the inputs and outputs. PCA uses the muscle activation matrix covariance to identify components that best describe the variance of the input data while minimizing the covariance of the basis vectors and constrains the components to be orthogonal (Abdi and Williams, 2010). While PCA accepts negative inputs, it constrains the synergies to be orthogonal, and this is not supported at any level in previous findings in experimentations with physiological systems that were modeled with PCA. This makes PCA very versatile and adaptable to negative data (such as kinematics) but probably not the best algorithm to describe neural control at the muscle level (Weiss and Flanders, 2004) because the components yielded using PCA impose a constrain that is not found at the physiological organization level and may not represent underlying “constructs.” In contrast, the underlying constructs can be labeled and readily interpreted using FA (Suhr, 2005). In a way similar to PCA, which tries to reproduce the total variable variance by a transformation of the input data, FA is also modeled as linear combinations of the factors and latent variables. However, FA is designed to identify a set of unobservable factors from the observed variables and attempts to reproduce the intercorrelations among variables, and the results were affected by the dependencies among activation coefficients (Tresch et al., 2006). In this study, the activation coefficients were from a set of experimental data that were randomly selected and grouped, which made the simulated muscle activations more independent and with few correlations among muscles. This might be one explanation that FA had a worse performance in reconstructing the variance of the muscle activations (VAF analysis) and quantifying spanned subspace (PA).

For the AEs, if only a single sigmoid hidden layer is used, the optimal solution is strongly related to PCA (Bourlard and Kamp, 1988; Chicco et al., 2014). The weights of AEs with a single hidden layer of size *p* (where *p* is less than the size of the input) span the same vector subspace as the one spanned by the first *p* principal components, and the output of the AE is an orthogonal projection onto this subspace. The potential of the AEs is their non-linearity, which allows the model to learn more powerful generalizations compared with PCA and FA and to reconstruct the input data with significantly lower information loss (Hinton and Salakhutdinov, 2006). From this perspective, the AEs are more appropriate for physiological signals analysis because they are usually non-linear and non-stationary.

In general, this study reported that NMF and ICA have better performance than the other methods. This finding is consistent with the abovementioned technical analysis of extraction methods. EMG signals are usually non-Gaussian data and exist with large non-linear and non-stationary components, which makes the NMF and ICA (non-Gaussian data input) outperform the PCA and FA (Gaussian data input) in muscle synergy extraction. Therefore, higher performance was observed when NMF and ICA were used to extract muscle synergies. Furthermore, our simulated data lack correlations among signals, thus FA lost priority in reproducing intercorrelations among variables. For the AEs, although some studies reported promising results in synergy extraction and force estimation using AEs (Spüler et al., 2016; Buongiorno et al., 2020), our results did not confirm this conclusion. One explanation is that AEs can achieve a trade-off between input space (muscle synergy extraction) and task space (force/moment reconstruction), while this study focused on synergy extraction not exploiting all the potential of this method. Moreover, several setups and design choices can be adopted for an AE, and the study did not test systematically all the possible configurations. Thus, even though AEs outperform FA and PCA in some settings, in our specific scenarios, NMF and ICA perform better.

Four synergies account for over 90% of the variability for NMF, ICA, and PCA in this study. Furthermore, 90% is often used as a “high” threshold to identify the optimal number of synergies in previous large studies, even though lower VAF values are used in some studies. Further analysis showed that VAF computed using FA was variable among different settings, and FA had the smallest VAF (78.91%) followed by AE (79.35%). This implies that, in our simulation, AE and FA could not capture the variance of muscle activation as well as the other methods when extracting the same number of synergies. On the contrary, NMF, ICA, and PCA had larger VAF among the five methods (98.53, 98.48, and 93.13%, respectively). The results were consistent with previous studies (Tresch et al., 2006; Ebied et al., 2018).

Similarity analysis first showed that all extraction methods had a better performance than random level except SVS of PCA. This exception can be explained as the structureless of the simulated data. In this study, randomly generated data were used to assess the performance of extraction methods, while muscles usually were activated in coordination manners. Some studies have used PCA to extract synergies from muscle activities and reported meaningful coordinated patterns among muscles.

Furthermore, the results showed that SNR and NoC had different influences on the performance of the extraction methods. In terms of SNR, all extraction methods performed better with the decrease of the noise (the increase of the SNR), i.e., SVS and ACS increased but PA decreased with the increase of the SNR, and differences were statistically significant. Similar results were reported in the previous work (Ebied et al., 2018; Kieliba et al., 2018). However, extraction methods had different performances when varying the NoC. First, SVS and ACS increased with the increase of the NoC for each extraction method. Similar results were observed for PA, especially for FA and AE. However, a high PA means worse performance. This indicates that PA has opposite performance with another two similarity indices. Remarkably, there was no statistical difference in the PA under different NoC settings for PCA, ICA, and NMF. Thus, we concluded that the performance of these three extraction methods (PCA, ICA, and NMF) is consistent under varying NoC. Second, the results showed that, when NoC was larger than 6Ch, SVS, ACS, and PA were less affected by NoC for PCA, ICA, NMF, and AE. The results suggest that, in the future muscle synergy analysis, it is not that more muscles are better. A limited number of muscles (eight muscles in this study) are enough to depict the variability of synergy structure.

In general, the results revealed that the noise and NoC affected the outputs of muscle synergy analysis, especially for noise. This has been proven by previous studies (Steele et al., 2013; Banks et al., 2017; Kieliba et al., 2018). They showed that signal preprocessing methods, synergy extraction methods, and the number and choice of muscles all affected the output of muscle synergies. AEs though showed better performance than FA in VAF and PCA in SVS and ACS, while losing priority compared with NMF and ICA. For the classification tasks, the results showed that synergies extracted from NMF and PCA had a higher classification accuracy, which indicates that these two extraction methods are suitable for classification tasks. In contrast, classification algorithms were sensitive to extraction methods. KNN and RF outperformed LDA and SVM in the current study for each extraction method. It suggests selecting the most suitable combinations of extraction methods and classification algorithms under different scenarios in future studies.

We did not investigate the influence of cross talk among muscles in this study. Cross talk influences the EMG signals and alters the components of muscle synergies. In this study, a publicly available dataset was used, in which the EMG signals were measured with Delsys double-differential EMG electrodes, and particular care was taken when placing the electrodes on the muscles (Atzori et al., 2014), which both reduce the influence of cross talk (Hug, 2011). Otherwise, previous studies showed that the number of independent motor command signals is not affected by cross talk, and muscle synergy analysis can identify whether a muscle is activated independently from an adjacent muscle even in the presence of cross talk (Chvatal and Ting, 2013). Besides, other synergy-related studies seldom considered the influence of cross talk while achieving simultaneous and intuitive myoelectric prosthetic control (Jiang et al., 2009, 2012, 2014; Zhang et al., 2017) and higher classification accuracy (Ma et al., 2015; Afzal et al., 2017). Thus, the results are limitedly influenced by cross talk in this study, so further studies are needed in the future.

Some limitations are worth noting. The study fixed the number of synergies to four. It is appropriate to study the dimension reduction capability of the extraction methods, and previous research pointed out that the number and choice of muscles impact the muscle synergy analyses (Steele et al., 2013). However, we also wish to mention that selecting a different number of synergies may lead to other sources of bias. In fact, if the very same method would be used for all algorithms to select the number of synergies (linear fit with a selected RMSE or selected VAF threshold), one may compare more “dense synergies” (when fewer synergies are extracted) to “more sparse ones,” leading to inconsistent matching because a different number of synergies were selected due to a very small amount of reconstruction of the overall variation. Finally, the transfer function and type of AEs may influence the performance of AEs in synergy extraction. In future studies, these problems will be further explored. We also plan to consider more settings to verify the feasibility and strength of these extracting methods in spatial synergy extraction.

## Conclusion

This article compared the performance of five muscle synergy extraction methods by the simulation analysis and classification tasks of a publicly available dataset. The results showed that the performance of synergy extraction methods was affected by the noise and NoC, and classification algorithms were sensitive to the extraction methods. Even though AEs outperformed FA and PCA in some settings, in general, NMF and ICA had better performance in the current research.

## Data Availability Statement

The original contributions presented in the study are included in the article/supplementary material, further inquiries can be directed to the corresponding author.

## Author Contributions

KZ, HW, and AS: conceptualization and methodology. KZ: software, validation, formal analysis, visualization, and writing-original draft. KZ and AS: investigation. KZ, MA, and HM: resources and data curation. HW, ZZ, MA, HM, and ZX: writing-review and editing. ZZ, HW, and AS: supervision and project administration. KZ, ZZ, and HW: funding acquisition. All authors provided intellectual contributions.

## Conflict of Interest

The authors declare that the research was conducted in the absence of any commercial or financial relationships that could be construed as a potential conflict of interest.

## Publisher’s Note

All claims expressed in this article are solely those of the authors and do not necessarily represent those of their affiliated organizations, or those of the publisher, the editors and the reviewers. Any product that may be evaluated in this article, or claim that may be made by its manufacturer, is not guaranteed or endorsed by the publisher.
